# RIP1/RIP3/MLKL-mediated necroptosis contributes to vinblastine-induced myocardial damage

**DOI:** 10.1007/s11010-020-03985-3

**Published:** 2020-11-28

**Authors:** Huiling Zhou, Lijun Liu, Xiaolong Ma, Jian Wang, Jinfu Yang, Xinmin Zhou, Yifeng Yang, Haidan Liu

**Affiliations:** 1 Department of Cardiovascular Surgery, The Second Xiangya Hospital of Central South University, Changsha, 410011 Hunan China; 2 Clinical Center for Gene Diagnosis and Therapy, The Second Xiangya Hospital of Central South University, Changsha, 410011 Hunan China

**Keywords:** Vinblastine, Necroptosis, Cardiotoxicity, MLKL

## Abstract

Vinblastine (VBL) has been considered as a first-line anti-tumor drug for many years. However, vinblastine-caused myocardial damage has been continually reported. The underlying molecular mechanism of the myocardial damage remains unknown. Here, we show that vinblastine induces myocardial damage and necroptosis is involved in the vinblastine-induced myocardial damage both in vitro and in vivo. The results of WST-8 and flow cytometry analysis show that vinblastine causes damage to H9c2 cells, and the results of animal experiments show that vinblastine causes myocardial cell damage. The necrosome components, receptor-interacting protein 1 (RIP1) receptor-interacting protein 3 (RIP3), are significantly increased in vinblastine-treated H9c2 cells, primary neonatal rat ventricular myocytes and rat heart tissues. And the downstream substrate of RIP3, mixed lineage kinase domain like protein (MLKL) was also increased. Pre-treatment with necroptosis inhibitors partially inhibits the necrosome components and MLKL levels and alleviates vinblastine-induced myocardial injury both in vitro and in vivo. This study indicates that necroptosis participated in vinblastine-evoked myocardial cell death partially, which would be a potential target for relieving the chemotherapy-related myocardial damage.

## Introduction

Cancer is a leading threat to human health all over the world [1]. Owing to the advances in anti-cancer therapies, the number of long-term cancer survivors has significantly increased. Meanwhile, the chemotherapy-induced cardiac complications including cardiotoxicity in patients receiving anti-cancer drug treatment also appear [2], which severely affect the life quality of patients. It is urgent clinical needs to elucidate the underlying mechanisms of chemotherapeutic agent-caused myocardial damage, optimize treatment strategies, minimize and finally prevent the risk of anti-cancer therapy-associated cardiovascular toxicities.

Emerging experimental and clinical data indicate that many anti-neoplastic drugs have side effects on the heart. Traditional chemotherapeutic drugs, such as platinum, doxorubicin, have historically been a major problem that causes cardiotoxicity. Molecular targeted therapy agents, including tyrosine kinase inhibitors (TKIs) lapatinib, sorafenib, imatinib and humanized monoclonal antibodies trastuzumab, bevacizumab, are known to cause cardiovascular toxic effects [3, 4]. The recent immunotherapeutic agents such as checkpoint inhibitors (e.g., ipilimumab, nivolumab and atezolizumab), though exhibiting a highly promising therapeutic effect with durable anti-cancer responses and long-term remission in a broad spectrum of cancers, have also been found to cause cardiac dysfunction [5]. The common cardiac toxic effects caused by anti-cancer therapies include left ventricular dysfunction (LVD), heart failure (HF), stoke, myocardial ischemia, myocarditis, cardiomyopathy [3–5] and so on. For example, platinum-based compounds can induce cardiac endothelial dysfunction, leading to myocardial ischemia and stoke [6]. Ipilimumab-caused LVD, cardiomyopathy or myocarditis has been reported. In addition, doxorubicin has been found to cause irreversible cardiotoxicity in 30–40% of patients, including massive myocytes loss, cardiomyopathy and heart failure. The occurrence of necroptosis is one of the important mechanisms for myocytes loss [7]. However, the underlying mechanisms of cardiotoxicity caused by many other chemotherapeutic drugs remain largely undisclosed.

Necroptosis is one type of the regulated cell deaths and is characterized by swelling of the cells, rupture of the plasma membrane and organelle dysfunction [8, 9]. Necroptosis can be elicited by members of the tumor necrosis factor receptor (TNFR) family (including FAS/CD95, TNFR1, TRAILR) [10] and interferon receptors [8]. Necroptosis can also be initiated by pathogen recognition receptors (PRRs), which include Toll-like receptors, NOD-like receptors as well as retinoic acid-induced gene Ι-like receptors [11, 12] and so on. Additionally, some anti-cancer drugs such as cisplatin and obatoclax have been shown to induce necroptosis [13]. Though the mechanisms of necroptosis execution are not fully understood, several signaling complexes that trigger necroptosis are progressively disclosed. Currently, the RIP1/RIP3 necrosome signaling complex is likely the most well established [14], whose formation can be induced by Fas, TNFR1, TRAILR and interferon-alpha receptor 1 (IFNAR1) [15]. Mechanistically, ligand-mediated receptor ligation leads to RIP1 binding to RIP3 via interacting with their RIP homotypic interaction motif (RHIM) domains [16]. RIP3 is then activated by auto-phosphorylation at Ser227 (human). The activated RIP3 recruits and subsequently phosphorylates MLKL at Thr357/Ser358 (human) [17]. Oligomerization occurs in the phosphorylated MLKL to form octamer, followed by the octamer translocating to intracellular and plasma membranes and destroying the integrity of cell [18, 19].

Accumulating evidence demonstrates that necroptosis is pathophysiologically relevant in many disease states, including stroke, myocardial infarction, atherosclerosis, ischemia reperfusion injury, neurodegenerative diseases, inflammatory diseases, infectious diseases and other clinically disorders [20]. Emerging studies indicate that necroptosis also participates in anti-tumor therapy-induced cardiac complications. For example, the inhibitors of RIP1 and RIP3 can alleviate dasatinib-induced cardiotoxicity [21]. However, the role of necroptosis in anti-tumor drug relevant cardiotoxicity has not been fully elucidated.

Vinca alkaloids, such as vinblastine, vinorelbine and vincristine, are anti-mitotic and anti-microtubule alkaloid agents derived from the periwinkle plant *Catharanthus roseus* [22]. Vinblastine is the first-line anti-neoplastic drug for treatment of Hodgkin’s lymphoma, choriocarcinoma and testicular tumors [23] and executes its cytotoxicity on cancer cells by binding to tubulin [24]. Since being used for clinical therapy, vinca alkaloid-caused heart damages have also been observed. For example, vinorelbine induces ST segment elevation myocardial infarction [25]. Vincristine causes coronary spasm [26] and cardiac arrest [27]. The cardiotoxicity induced by vinca alkaloids is mainly due to their causing degeneration and death of myocardial cells [28]. However, the mechanistic detail between vinca alkaloids, including vinblastine, and death of myocardial cells still remains largely unknown.

Here, we investigate the underlying mechanism of vinblastine-induced cardiotoxicity. We demonstrate that vinblastine damages cardiomyocytes in vitro and in vivo. Mechanism study demonstrates that vinblastine promotes the expression of necroptosis-associated proteins in cardiomyocytes. And Nec-1, the inhibitor of RIP1, attenuates the vinblastine-induced cardiotoxicity in vitro and in vivo. The study reveals that necroptosis, at least in part, contributes to vinblastine-induced cardiomyocytes death. The findings provide insight into a possible molecular mechanism underlying chemotherapeutic agent-caused cardiotoxicity and suggest necroptosis pathways might be potential targets for protecting myocardial cells from anti-tumor-induced cardiac injury.

## Materials and methods

### Reagents

Vinblastine sulfate (>99% purity, cat#HY-13780), daunorubicin (cat#HY-13062), doxorubicin (cat#HY-15142) and necrostatin-1 (cat#HY-15760) were purchased from MedChemExpress (New Jersey, USA). 3-Methyladenine (cat#M9281), cisplatin (cat#479306), 5-Fluorouracil (cat#F6627), 2,3-Butanedione monoxime (BDM, cat#B0753), Trypsin-EDTA (0.25%) (cat#25200056), Bromo-2′-deoxyuridine (BrdU, cat#B5002) and chemical reagents, including Tris, SDS, DMSO and NaCl, for molecular biology and buffer preparation, were purchased from Sigma-Aldrich (St. Louis, MO, USA). Collagenase Type II (cat#LS004176) was purchased from Worthington (New Jersey, USA). GSK’872 (cat#S8465), Necrosulfonamide (cat#S8251), SB203580 (cat#S1076), BV6 (cat#S7597), ZVAD-fmk (cat#S7023) were purchased from Selleckchem (Houston, TX, USA). TNFα (cat#AF-300-01A) was purchased from Peprotech (Rocky Hill, USA). DMEM/F12 (cat#11765054) and Medium 199 (M199, cat#11150059) were purchased from Thermo Fisher Scientific (Waltham, USA). D-Hanks (cat#BL559A) was purchased from Biosharp (Hefei, China). Falcon Cell Strainers (cat#352360) was purchased from Coining (New York, USA).

### Cell line and cell culture

The rat embryonic ventricular myocardial cell line H9c2 was purchased from Cell Bank of Chinese Academy of Sciences (Shanghai, China). Cells were cultured in Dulbecco’s modified Eagle’s medium (DMEM) containing 1.5 g/L NaHCO_3_, supplemented with 10% fetal bovine serum (FBS) and 1% antibiotics at 37 °C in a humidified incubator with 5% CO_2_. FBS (cat#04-001ACS) was purchased from Biological Industries (Israel). Each vial of frozen cells was thawed and maintained for less than 10 passages.

### Primary neonatal rat ventricular myocytes isolation and culture

Neonatal rat ventricular myocytes were prepared from 1-to 2-day-old Sprague-Dawley rat. Briefly, neonatal rat ventricles were dispersed in a series of incubations in isolation medium containing 20 mM BDM, 0.0125% trypsin and D-Hanks at 4 °C overnight. Then the supernatant was removed and digestion medium, containing 20 mM BDM, 1.5 mg/mL collagenase type II and DMEM/F12, and 5 mL of DMEM/F12 at 37 °C for 30 min, was added, and then supernatant was transferred to a cell strainer. Cells were centrifuged and the supernatant was removed. Cells were re-suspended in plating medium containing 0.1 mM BrdU, 65% DMEM, 19% M199, 15% FBS and 1% antibiotics for 18 h, and then cultured at 37 °C in a humidified incubator with 5% CO^2^ in maintenance medium containing 0.1 mM BrdU 78% DMEM, 17% M199, 4% FBS and 1% antibiotics for 5 days.

### Cell viability assay

Cell viability was assessed using the cell counting kit-8 (CCK-8, cat#B34302, Bimake, USA) according to the manufacturer’s instructions. Briefly, cells were seeded at a density of 3 × 10^3^ cells per well in 96-well plates in 100 μL culture medium without or with various concentrations of vinblastine and different types of inhibitors, and then incubated in a 37 °C, 5% CO^2^ incubator. After culturing for 0, 24 or 48 h, 10 μL of the CCK-8 reagent was added to each well and cells were incubated for 2 h at 37 °C. The absorbance of the sample was measured by EL × 800 microplate reader (Biotek Instruments Inc., Vermont, USA.) at 450 nm as previously described [29]. At least two independent experiments were performed in triplicate.

### Flow cytometry analysis

Flow cytometry assay was performed as described previously [30]. In brief, cells were seeded in 6-well plates in 2 mL of medium at a 37 °C, 5% CO^2^ atmosphere. After 48 h of vinblastine treatment, cells were harvested and then incubated with 300 μL binding buffer, 3 μL Annexin V-FITC and 6 μL propidium iodide in the dark for 10 min at room temperature. Stained cells were then analyzed using FacsCantoTM II flow cytometer (BD, Biosciences, CA, USA). At least two independent experiments were performed in triplicate.

### Western blot analysis

H9c2 cells and primary neonatal rat ventricular myocytes were harvested after treatment for 48 h and lysed in RIPA lysis buffer (cat#89900, Thermo scientific, Rockford, IL, USA) containing protease inhibitor cocktail (Roche, Mannheim, Germany) and phosphatase inhibitors (Roche, Mannheim, Germany), and then kept on ice for 30 min. After that, the supernatant was collected and centrifuged at 12,000 rpm for 10 min at 4 °C. Protein concentrations were measured by BCA Assay Reagent (cat#23228; Pierce, Rockford, IL, USA), and then the extracts stored at −80 °C as previously described [31]. Equal amount of samples were resolved by SDS-PAGE, transferred to PVDF membrane and probed at 4 °C overnight with the following antibodies: RIP1 (#3493), phospho-CaMKII (Thr286) (#12716; Note: this antibody can also recognize endogenous levels of CaMKII-β and CaMKII-γ protein only when phosphorylated at Thr287, according to the product description), CaMKII (pan) (#4436) from Cell Signaling Technology, RIP3 (#NBP1–77299) from Novus Biologicals (Minneapolis, MS, USA), MLKL (#orb32399) from Biorbyt (Cambridge, Britain), Anti-oxidized-CaMKII (Met281/282) (#07–1387) from Millipore (Burlington, MA, USA), and GAPDH (#5G4-6C5, HyTest Ltd., Turku, Finland). The blots were incubated for 45 min at room temperature with horseradish peroxidase (HRP)-conjugated anti-rabbit (sc-2004, Santa Cruz Biotechnology) or anti-mouse (sc-2005, Santa Cruz Biotechnology) IgG. Protein bands were visualized with a chemiluminescent substrate (ECL; cat#34076, Thermo Scientific).

### Co-immunoprecipitation (Co-IP) assay

H9c2 cells were treated with DMSO or the indicated concentrations of vinblastine for 48 h. Cells were harvested in IP lysis buffer (Cat. No.87788, Thermo Scientific). Cell extracts were pre-cleared with 40 μL of protein A/G-agarose beads (sc-2003, Santa Cruz Biotechnology), and immunoprecipitated with 2 μg of anti-RIP3 (cat#95702, Cell Signaling Technology) or normal rabbit IgG (cat#NI01, Calbiochem) at 4 °C overnight, and then incubated with 40 μL of proteins A/G-agarose beads at 4 °C for 2 h as described previously [32]. Immunocomplexes were resolved by SDS-PAGE and co-immunoprecipitated protein was disclosed using RIP1 and MLKL, respectively.

### Animal experiments

Male Sprague-Dawley (SD) rats (200 g–220 g) were purchased from Hunan SJA Laboratory Animal Co., Ltd. All animal experiments were approved by the Animal Ethics Committee of Central South University, Hunan Province, China and animal experiments followed the PREPARE (Planning Research and Experimental Procedures on Animals: Recommendations for Excellence) guidelines. Animals were maintained under specific pathogen-free (SPF) conditions and housed in individual ventilated cages, kept 3 rats per cage on a 12 h light/dark cycle in a temperature-controlled (21 ± 2 °C) room with free access to water and food. The rats were allowed 5 days to adapt to the laboratory environment. Animals were randomly assigned to the following groups: control group (*n* = 12), VBL group (*n* = 19) and VBL + Nec-1 group (n = 19). For the control group, the animals were injected with saline. For the VBL group, the animals were administered 1.3 mg/kg vinblastine every three days via tail vein for a total of six injections. Rats in the VBL + Nec-1 group received 1.5 mg/kg Nec-1 30 min in advance, followed by the injection of 1.3 mg/kg vinblastine via tail vein. Four hours after the final injection, all animals were sacrificed, and hearts were removed, weighed and photographed.

### Hematoxylin and eosin (HE) staining

Rat hearts were removed and fixed immediately using 10% formaldehyde solution for 24 h and sliced into 4-μm-thick sections. Subsequently, the sections were stained with hematoxylin and eosin for histological analysis. Images were photographed under a microscope (Leica. Germany).

### Immunohistochemistry (IHC) staining

The paraffin-embedded rat left ventricular tissues were sectioned, deparaffinized and boiled in sodium citrate buffer for 10 min for antigen retrieval, and then blocked with 5% BSA for 30 min at room temperature as described previously [29]. Immunohistochemistry staining were performed with antibodies against RIP1 (1:100, ab72139, Abcam), RIP3 (1:150, NBP1–77299, Novus), MLKL (1:100, orb32399, Biorbyt) according to the manufacturer’s instructions. Hematoxylin was used for counterstaining. All sections were observed by microscope (100×) and analyzed the mean density of photographs using Image-Pro Plus (version 6.0) software program.

### Statistical analysis

Statistical analysis was performed with SPSS 16.0 (SPSS, Inc., Chicago, IL). Results expressed as mean ± SD were analyzed using the Student’s *t* test. Survival curve was estimated using the Kaplan–Meier method. The log-rank test was used to identify statistically significant differences between survival curves. Differences were considered significant when *p* < 0.05.

## Results

### Vinblastine inhibits rat H9c2 myocardial cell viability

**Fig. 1:**
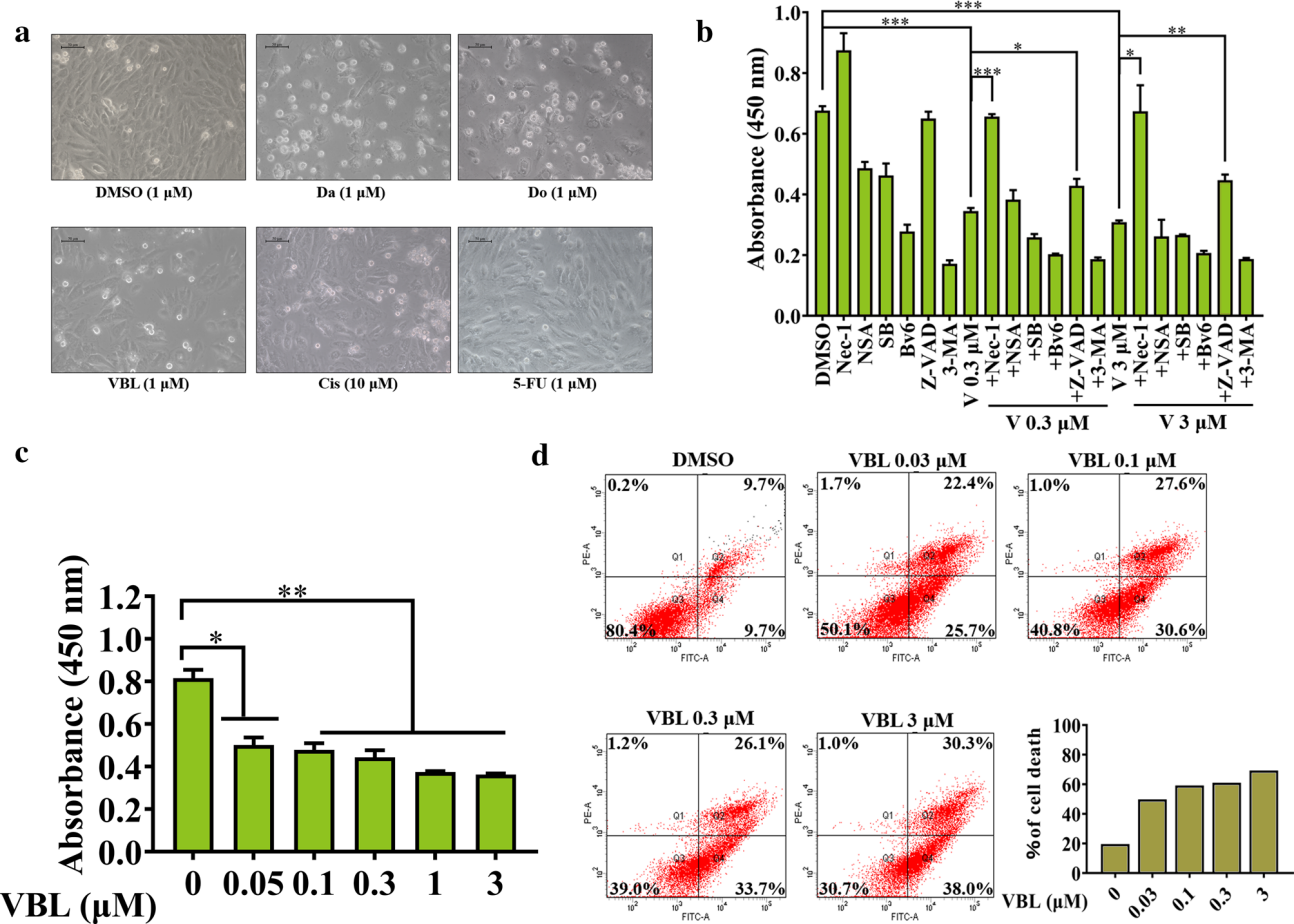
Effect of vinblastine on the viability of H9c2 cells. (**a**) H9c2 cells were treated with DMSO, daunorubicin (Da), doxorubicin (Do), vinblastine (V), cisplatin (Cis) and 5-fluorouracil (5-Fu) at the indicated concentrations for 48 h and were observed under a microscope (200 ×). (**b**) H9c2 cells were pre-treated with 10 μM SB203580 (SB), 5 μM BV6, 50 μM ZVAD-fmk or 5 mM 3-MA for 1 h and treated with DMSO or the indicated concentrations of vinblastine for 48 h. Cell viability was measured using the WST-8 assay. Data represent mean ± SD from three independent experiments. *p < 0.05, **p < 0.01, ***p < 0.001. (**c**) H9c2 cells were treated with DMSO or various concentrations of vinblastine for 48 h and cell viability was measured by WST-8 assay. Data represent mean ± SD from three independent experiments. *p < 0.05, **p < 0.01 vs. DMSO-treated group. (**d**) H9c2 cells were treated with DMSO or vinblastine (0.01, 0.03, 0.3 or 3 μM) for 48 h; cells were subjected to propidium iodide and Annexin V-FITC staining and analyzed by flow cytometry

To assess the toxic effect of chemotherapeutic agents on cardiomyocytes, rat H9c2 myocardial cells were treated with clinical anti-tumor drugs, including doxorubicin, daunorubicin, vinblastine, cisplatin and 5-fluorouracil. We found that doxorubicin, daunorubicin and vinblastine caused a significant cell death of H9c2 at 1 μM (Fig. 1a). Moreover, we found that necroptosis inhibitor Nec-1 [33] can partially decrease vinblastine-induced H9c2 cell death (Fig. 1b), while doxorubicin and daunorubicin cannot (data not shown), suggesting that necroptosis contributes to vinblastine-induced H9c2 cell death. We thus focus on vinblastine for study. When further treated H9c2 cells with different concentrations of vinblastine, we found that vinblastine dose-dependently caused cell death (Fig. 1c and d). These results suggest that vinblastine inhibits rat H9c2 myocardial cell viability.

### Vinblastine causes rat myocardial injury in vivo

**Fig. 2:**
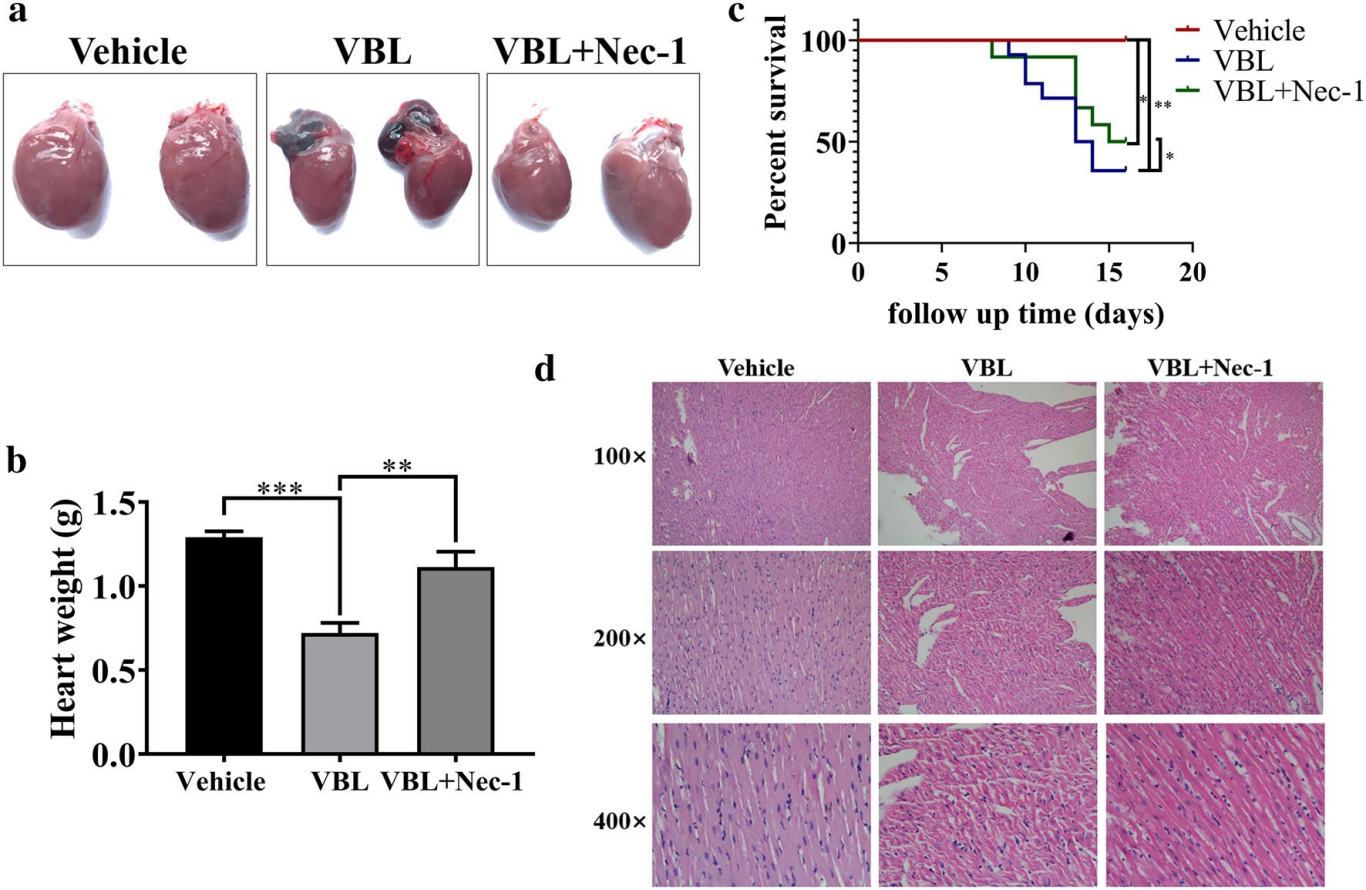
Vinblastine causes rat myocardial injury in vivo. (**a**) SD rats were randomly divided into three groups and treated as described in Materials and Methods. At the treatment endpoint, rats were sacrificed and hearts were removed and photographed. (**b**) Hearts of rats were weighed and recorded. **p < 0.01, ***p < 0.001. (**c**) Overall survival rates of SD rats with saline (*n* = 7), VBL (*n* = 14) or VBL+ Necrostatin-1 (Nec-1) (*n* = 12) were analyzed with Kaplan–Meier method by log-rank test. *p < 0.05, **p < 0.01. (**d**) H&E staining of heart tissues from the rats of saline-, VBL- and VBL + Nec-1-treated groups. Figures were representative of two independent experiments

To evaluate the toxic effect of vinblastine on heart in vivo, Sprague-Dawley rats were administered vinblastine via tail vein. The results showed that the size and weight of vinblastine-treated hearts were smaller and lower than those of the vehicle-treated group (Fig. 2a and b), indicating that the loss of cardiomyocytes upon vinblastine treatment. Vinblastine also caused right atrial congestion (Fig. 2a). Additionally, the survival time of SD rats in vinblastine-treated group was significantly shorter than that of the vehicle-treated group (Fig. 2c). HE staining was utilized to visualize the myocardial injury in vinblastine-treated SD rat model. Compared with the vehicle-treated group, it was obvious that the disordered arrangement of myocardial myofibrils and widened muscle gap in the vinblastine-treated group. Besides, red blood cells in the myocardial interstice were observed, suggesting that vinblastine may also damage blood vessels (Fig. 2d). Notably, pre-treated with necroptosis inhibitor Nec-1 significantly alleviated vinblastine-induced heart weight loss and atrial congestion (Fig. 2a and b), improved the survival time (Fig. 2c), and recovered myocardial myofibril from irregular arrangement induced by vinblastine (Fig. 2d). These results suggest that vinblastine leads to cardiomyocytes injury in vivo.

### Necroptosis is involved in vinblastine-induced myocardial damage

**Fig. 3:**
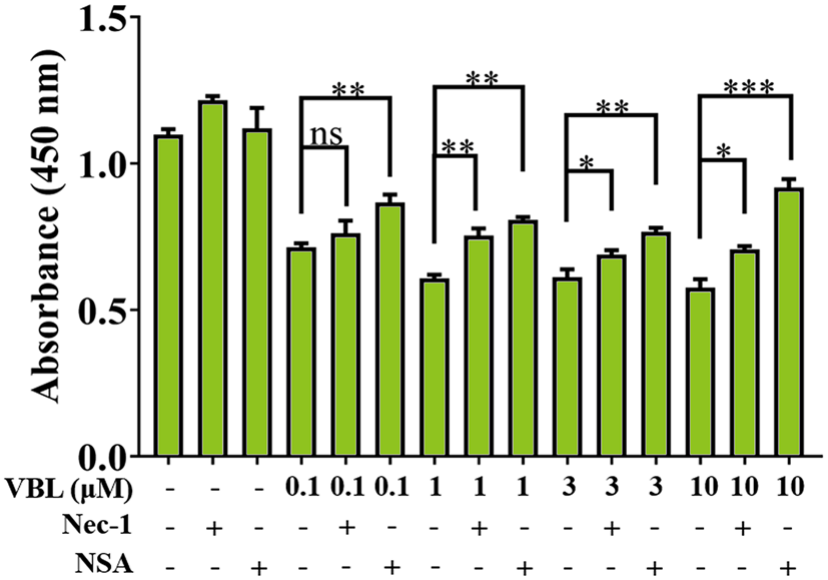
Necroptosis is involved in vinblastine-induced H9c2 cell death. The inhibitors of necroptosis ameliorate the VBL-induced cardiomyocytes injury. H9c2 cells were pre-treated with 50 μM Necrostatin-1 (Nec-1) or 2.5 μM Necrosulfonamide (NSA) for 1 h and then treated with DMSO or the indicated concentrations of VBL for 48 h. Cell viability was measured using the WST-8 assay. Data represent mean ± SD from three independent experiments. *p < 0.05, **p < 0.01, ***p < 0.001

Our results showed that necroptosis inhibitor Nec-1 significantly alleviated vinblastine-caused myocardial injury both in vitro and in vivo (Figs. 2 and 1b), suggesting that necroptosis is involved in vinblastine-induced myocardial cell death. We further treated H9c2 cells with MLKL inhibitor NSA [34] and observed that both Nec-1 and NSA significantly improved the viability of vinblastine-treated H9c2 cells detected by WST-8 assays (Fig. 3). These results reinforce the hypothesis that necroptosis, at least in part, is involved in vinblastine-induced myocardial cell death and finally causes myocardial damage.

### RIP1/RIP3/MLKL-mediated necroptosis contributes to vinblastine-induced myocardial damage

**Fig. 4:**
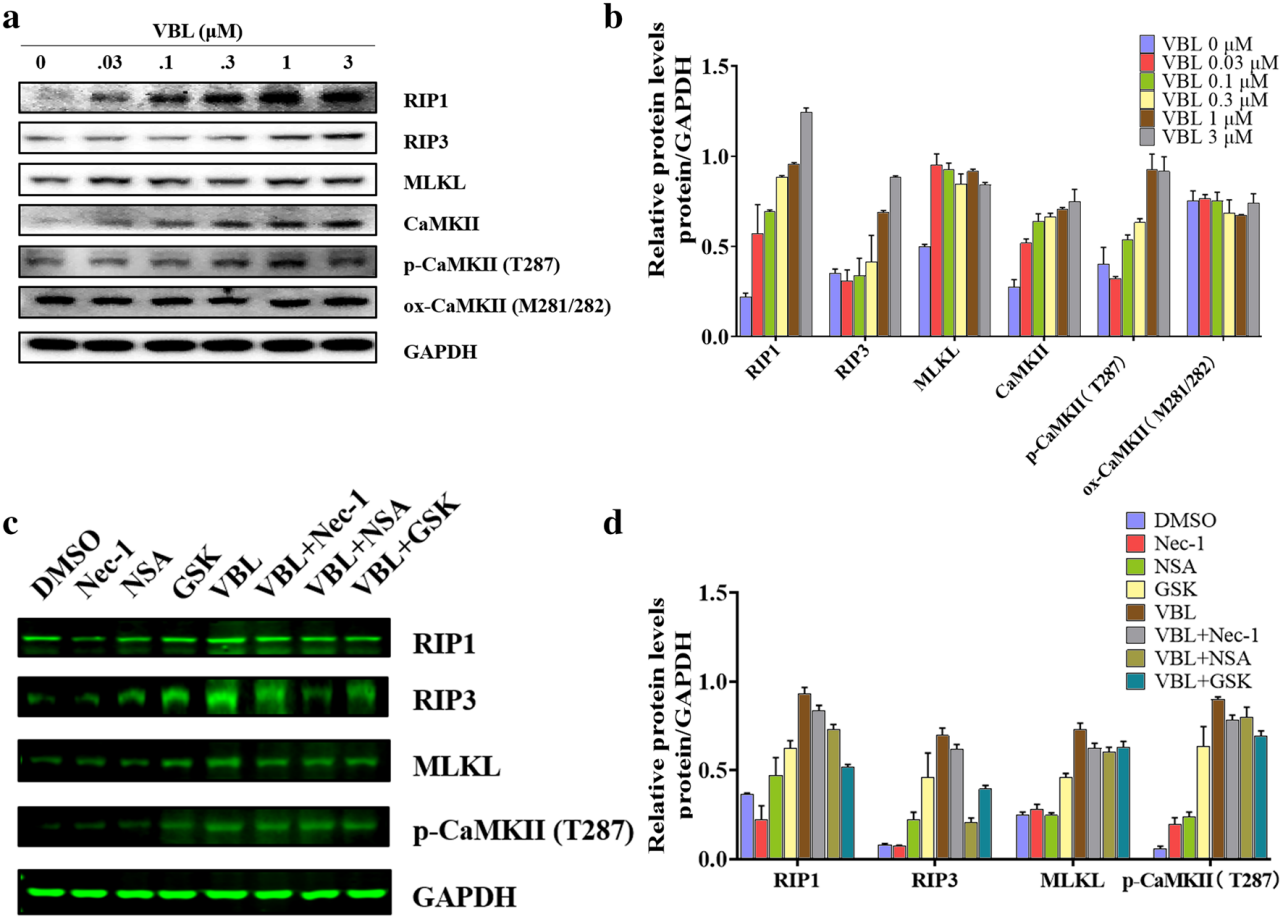
Effect of vinblastine on the expression of necroptosis-related proteins in H9c2 cells. (**a**) H9c2 cells were treated with DMSO or the indicated concentrations of vinblastine for 48 h. After treatment, the attached and floating cells were harvested. Expression of the indicated proteins was analyzed by Western blotting with specific antibodies. GAPDH was used as a loading control. (**b**) The relative protein level of each protein was shown. (**c**) H9c2 cells were pre-treated with the indicated necroptosis inhibitors for 1 h and then treated with DMSO or 0.3 μM vinblastine for 48 h. After treatment, the attached and floating cells were harvested. Expression of the indicated proteins was analyzed by Western blotting with specific antibodies. GAPDH was used as a loading control. (**d**) The relative level of each protein was shown. Data are representative of three independent experiments

**Fig. 5:**
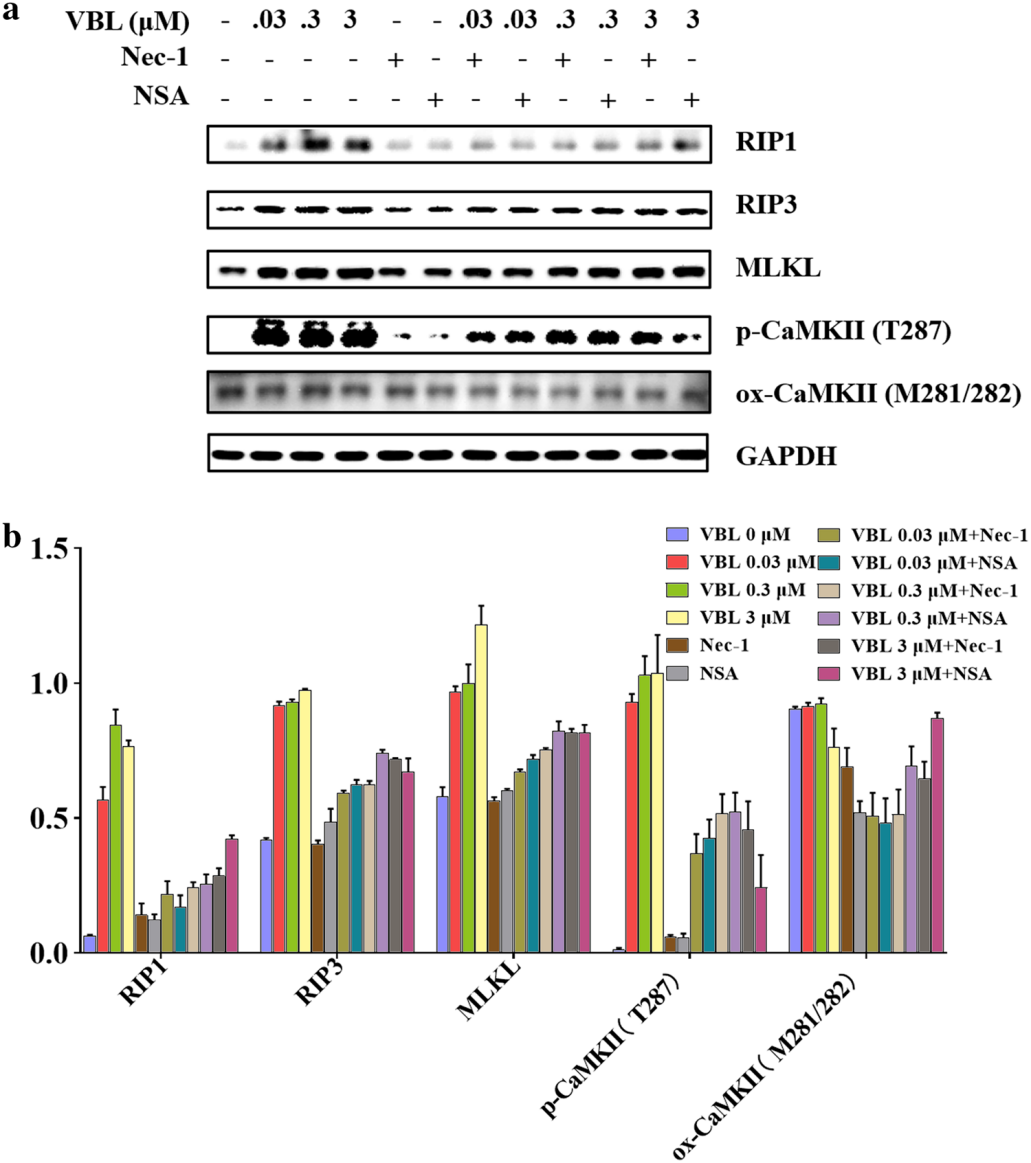
Effect of vinblastine on the expression of necroptosis-related proteins in primary neonatal rat ventricular myocytes. (**a**) Primary neonatal rat ventricular myocytes were pre-treated with indicated inhibitors for 1 h and treated with DMSO or indicated concentrations of vinblastine for 48 h. Expression of the indicated proteins was analyzed by Western blotting with specific antibodies. GAPDH was used as a loading control. (**b**) The relative protein level of each protein was shown. Data are representative of two independent experiments

To further confirm that necroptosis is involved in vinblastine-induced myocardial damage, the necroptosis-related proteins were investigated in vinblastine-treated H9c2 cells by Western blotting. The results indicated that the expressions of necrosome components RIP1, RIP3 were increased upon vinblastine treatment, and the substrate of RIP3, MLKL was also increased (Fig. 4a and b). Subsequently, H9c2 cells were pre-treated with Nec-1, NSA or RIP3 inhibitor GSK’872 [35], and then exposed to vinblastine. Western blotting results demonstrated that Nec-1, NSA and GSK’872 partially inhibited vinblastine-increased RIP1, RIP3 and MLKL, respectively. Notably, inhibition each of the necrosome components by their respective inhibitor also resulted in the downregulation of the other two components (Fig. 4c and d), suggesting that the protein level of each necrosome component is dependent on the presence of the other members of the complex. Further, we isolated the primary neonatal ventricular myocytes from SD rat, and could observe cells pulsating under the microscope (data not shown). To verify whether the necroptosis involved in neonatal rat ventricular myocytes treated with vinblastine, we utilized Western blotting to investigate the expression of necroptosis-related proteins. The results shown that the vinblastine augmented the expression of RIP1, RIP3 and MLKL in primary myocytes. Concurrently, ventricular myocytes were pre-treated with Nec-1 and NSA, and the WB results manifested that these inhibitors alleviated the increase in necroptosis-related proteins induced by vinblastine (Fig. 5). Taken together, these results support the assumption that RIP1/RIP3/MLKL-mediated necroptosis, at least partially, is the mechanisms of vinblastine-induced cardiomyocyte cell death, which finally results in myocardial damage.

### Vinblastine promotes the formation of RIP1/RIP3-containing necrosome in H9c2 cells

**Fig. 6:**
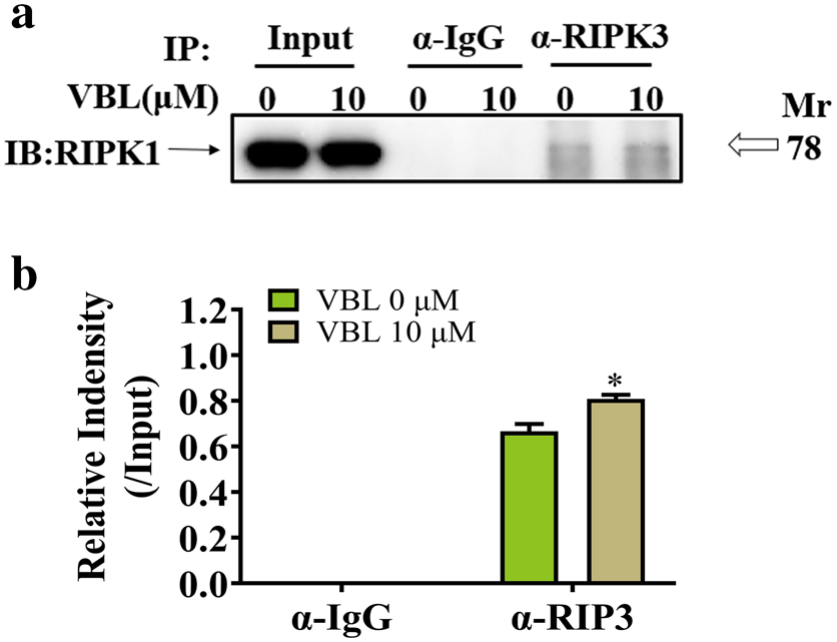
Vinblastine promotes the endogenous RIP1 and RIP3 interaction. (**a**) H9c2 cells were cultured with DMSO or the indicated concentrations of vinblastine for 48 h. After treatment, cells were lysed and immunoprecipitated with a RIP3 or a normal IgG antibody. The immune complexes and input were analyzed by immunoblotting with a RIP1 antibody. (**b**) The data were normalized to input and shown as mean ± SD of two separate experiments. *p < 0.05, significant difference compared with the DMSO-treated group

The formation of RIP1/RIP3-containing necrosome plays a crucial role in necroptosis execution [36]. In order to determine whether treatment of vinblastine could affect necrosome assembly, we immunoprecipitated RIP3 from H9c2 cells treated without or with vinblastine, and RIP1 was probed by Western blotting. The co-IP data indicated that compared with the DMSO control, treatment of vinblastine significantly increased interaction of RIP3 and RIP1 (Fig. 6a and b). The result implies that vinblastine induces the formation of RIP1/RIP3-containing necrosome in H9c2 cells.

### RIP1/RIP3/MLKL-mediated necroptosis contributes to vinblastine-induced rat myocardial damage in vivo

**Fig. 7:**
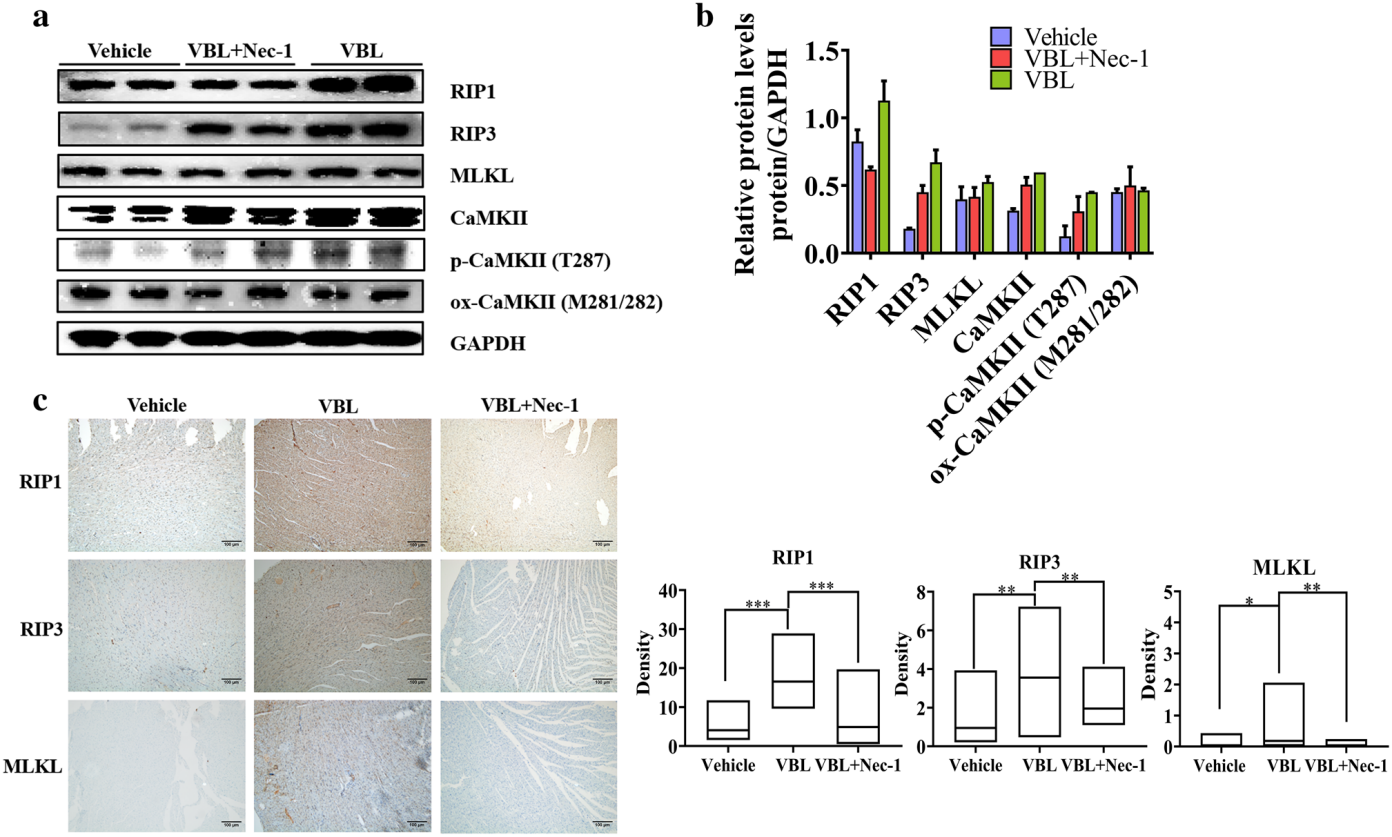
Effect of vinblastine on the expression of necroptosis-related proteins in SD rats. (**a**, **b**) SD rats were randomly divided into three groups and treated according to the protocol described in Materials and Methods. At the treatment endpoint, rats were sacrificed and left ventricle tissues were collected and lysed. Expression of the indicated proteins was analyzed by Western blotting with specific antibodies. GAPDH was used as a loading control. (**c**) Immunohistochemical staining examination of RIP1, RIP3 and MLKL in rat left ventricle tissues from the saline-, VBL- or VBL + Nec-1-treated group. All panels were shown as the indicated magnification (100×). And the density of each protein was shown. *p < 0.05, **p < 0.01, ***p < 0.001. Data were representative of two independent experiments

In order to confirm the role of necroptosis in vinblastine-induced myocardial damage in vivo, we checked the relevant proteins of necroptosis in rat ventricular tissues by Western blotting. Compared with the vehicle group, the levels of RIP1, RIP3 and MLKL in vinblastine-treated group were augmented (Fig. 7a and b). Pre-treatment with Nec-1 decreased not only vinblastine-augmented RIP1, but also vinblastine-augmented RIP3, MLKL (Fig. 7a and b), which were consistent with the cell-based results described above (Fig. 4). The IHC staining results indicated that vinblastine caused significant increases of RIP1, RIP3 and MLKL in rat ventricular tissues, whereas pre-administration of Nec-1 remarkably inhibited vinblastine-caused RIP1, RIP3 and MLKL elevations (Fig. 7c), which coincided with the Western blotting results (Fig. 7a), suggesting that Nec-1 prevents vinblastine-caused myocardial damage by inhibiting the RIP1/RIP3/MLKL-containing necrosome assembly. Collectively, the results re-confirm that MLKL-mediated necroptosis, at least partially, is the underlying mechanisms of vinblastine-induced cardiomyocyte cell death, which finally results in myocardial damage.

## Discussion

In the present study, we observed that the vinca alkaloid anti-cancer drug vinblastine, significantly injured cardiomyocytes both in vitro and in vivo. We also revealed that necroptosis was involved in vinblastine-caused myocardial cell death, and the necroptosis inhibitors could ameliorate vinblastine-induced myocardial damage both in vitro and in vivo.

Our co-IP result showed that vinblastine promoted the interaction of RIP1 with RIP3 (Fig. 6). Moreover, vinblastine treatment elevated the expression of necrosome components RIP1, RIP3 and MLKL both in cell culture and animal models (Figs. 4, 5 and 7). From these results combined, we speculated that vinblastine increases the formation of RIP1/RIP3-containing necrosome, which plays a role in vinblastine-induced myocardial injury.

Our echocardiography results showed no significant differences among three groups (date not shown). The possible reasons may be (1) the activation of cardiac reserves and compensatory mechanisms. (2) vinblastine-induced cardiotoxicity is a dose accumulation process. Short-term vinblastine treatment may cause early cardiotoxicity, which might be accompanied by only pathological change without organic lesions. The appearance of an obvious contractile dysfunction might need a long-term doxorubicin exposure to induce chronic cardiotoxicity [37].

Growing evidence has proved that MLKL is dispensable for necroptosis. Chang et al. discovered that MLKL does not impact the RIP3-induced necroptosis in acute myocardial infarction [38]. Sun et al. reported that CaMKII (calcium/calmodulin-dependent kinase II) rather than MLKL participates in high glucose-induced cardiomyocytes injury [39]. CaMKII is enriched in myocardium, which is a multifunctional Ser/Thr protein kinase consisting of four isoforms (α, β, δ and γ) [40]. CaMKII activation is primarily achieved through two pathways: phosphorylation at Thr287 [41] and oxidation at Met281/281 [42]. Activated CaMKII results in dissipation of inner mitochondrial membrane potential (ΔΨm) and indirect reactive oxygen species (ROS)-mediated intracellular Ca^2+^ alterations finally induce cell death [43]. Recently, CaMKII is identified as a novel substrate of RIP3. This study revealed that RIP3 directly phosphorylates CaMKII at Thr287 for its activation, and contributes to both ischemic/reperfusion (I/R)- and doxorubicin-induced myocardial necroptosis [7]. Similar result that RIP3 directly binds and phosphorylates CaMKII at Thr287 was reported by other researchers [39, 44]. In addition, Qu et al. found that RIP3 interacts with MLKL and CaMKII to mediate oligodendrocytes necroptosis in the development brain [45]. Yang et al. reported that both MLKL and CaMKII involved in chronic pain-associated myocardial ischemia which RIP3 evoked [44]. These studies suggested that multi-mechanisms exist for necroptosis execution, which might depend on different cell types. Our results showed that vinblastine increased the levels of RIP1, RIP3 and MLKL as well as CaMKII and phospho-CaMKII (Thr287) both in vitro and in vivo (Figs. 4, 5 and 7), suggesting that vinblastine-induced cardiomyocytes necroptosis might be triggered by not only MLKL-mediated but also CaMKII-mediated cascades, but it requires further investigation.

Our results shown that the levels of CaMKII and phospho-CaMKII were increased by vinblastine treatment in H9c2 cells (Fig. 4a and b). By using a site-specific antibody to detect ROS-induced oxidation of CaMKII, the methionine oxidation of CaMKII at Met281 and Met282 (Met281/282) remained no obvious change after vinblastine treatment in H9c2 cells (Fig. 4a and b). Additionally, the levels of CaMKII and phospho-CaMKII were also increased in both vinblastine-treated primary neonatal rat ventricular myocytes (Fig. 5) and rat heart tissues (Fig. 7). Pre-treatment with Nec-1 decreased not only vinblastine-augmented RIP1, but also vinblastine-augmented CaMKII and phospho-CaMKII in both primary neonatal rat ventricular myocytes (Fig. 5) and rat heart tissues (Fig. 7a and b) However, an earlier report showed that phosphorylation of CaMKII at Thr287 and CaMKII oxidation at Met281/282 were increased upon doxorubicin treatment and both contribute to doxorubicin-mediated myocardial necroptosis [7]. We speculated that different chemotherapeutic drugs caused cardiotoxicity through diverse mechanisms. Whether CaMKII is involved in vinblastine-induced myocardial damage needs to be further explored.

When pre-treated with apoptosis inhibitor (ZVAD-fmk), autophagy inhibitor (3-MA) or necroptosis inhibitors (Nec-1 and NSA) and analyzed the effects on the vinblastine-treated H9c2 cells, our results showed that besides necroptosis inhibitors, the pan-caspase inhibitor ZVAD-fmk could partially rescue the injury induced by vinblastine (Fig. 1b), suggesting that apoptosis may also involve in the myocardial damage by vinblastine treatment. Similar to our results, it has been reported that both necroptosis and apoptosis contribute to myocardial damage caused by doxorubicin, a frontline chemotherapeutic agent [7]. Takayuki Kimura et al. found that the inhibition of autophagy pathway significantly attenuated sunitinib-induced cytotoxicity in rat H9c2 cardiomyocytes and human-induced pluripotent stem cell-derived cardiomyocytes, suggesting autophagy is involved in sunitinib-induced cardiotoxicity [46]. In our present study, the relative contributions of necroptosis and apoptosis to vinblastine-induced cardiac damage need to be further evaluated.

In fact, vinblastine is not the first anti-neoplastic drug to be found to cause myocardial injury. Since the first report concerned myocardial injury of anthracycline in 1976, through long-term monitoring and follow-up of more than 60,000 elderly patients with early-stage breast cancer in the United States, the researchers discovered that cardiovascular events were the leading cause of death (15.9%) in breast cancer patients aged >66 years old [47]. Since then, a plethora of anti-neoplastic drugs were discovered to cause varying degrees and types of cardiovascular damage. These phenomena led researchers to draw attention to anti-neoplastic drug-associated cardiotoxicity. An innovative interdisciplinary subject, the onco-cardiology was established. Onco-cardiology commits to study the occurrence of cardiovascular disease caused by the tumor and anti-tumor treatment. Many mysteries are still waiting to be unveiled by both cardiologists and oncologists for the optimal care of cancer patients.
